# Assessing Protective Factors of Youth Who Sexually Offended in Singapore: Preliminary Evidence on the Utility of the DASH-13 and the SAPROF

**DOI:** 10.1177/1079063214561684

**Published:** 2015-02

**Authors:** Gerald Zeng, Chi Meng Chu, Yirong Lee

**Affiliations:** 1 Ministry of Social and Family Development, Singapore

**Keywords:** juvenile sex offender, juvenile sex offender recidivism, protective factors, desistence

## Abstract

Sexual offending has attracted increasing public concern because of its long-term effects. Although there is an increasing amount of research on the risk factors for recidivism among youth who have sexually offended, there is a dearth of research on the protective factors for desistence from recidivism. The current study investigated the associations between protective factors and recidivism among 97 Singaporean youth who sexually offended (YSO). In addition, the predictive validity with regard to two new measures of protective factors—the Desistence for Adolescents Who Sexually Harm (DASH-13), and Structured Assessment of Protective Factors for Violence Risk (SAPROF)—were also evaluated. Results indicated that both the DASH-13 and the SAPROF were inversely related to the Estimate of Risk of Adolescent Sexual Offense Recidivism (ERASOR). However, neither the DASH-13 nor the SAPROF were found to have adequate predictive validity or incremental validity for sexual or nonsexual recidivism. The implications for the assessment and management of YSO are discussed.

## Introduction

Sexual offending is highly intrusive and is linked to many adverse long-term effects for the victims (Campbell & Wasco, 2005; Noll, Horowitz, Bonanno, Trickett, & Putnam, 2003). There has been increasing public concern about the risk of recidivism among youth who sexually offended (YSO) and these concerns have led to restrictive policies and clinical practices (Garfinkle, 2003; Worling & Långström, 2006). Mental health clinicians are often required to conduct risk assessments for YSO, with the assumption that the clinicians can accurately predict the risk of recidivism in these youth, but this may not necessarily be the case (Caldwell, Ziemke, & Vitacco, 2008). Notwithstanding that there is a greater focus on understanding youth sexual offending issues in recent years (see Seto & Lalumière, 2010 for a review), there is a relative paucity of empirical research on the protective factors that buffer YSO against recidivism within a non-Western context.

### Conceptualization of Protective Factors for Antisocial and Offending Behaviors

In the study of antisocial or offending behaviors, risk factors are defined as factors that are associated with the higher probability of a negative outcome (e.g., violent or sexual recidivism; Lösel & Farrington, 2012). However, protective factors lack a standardized definition and can be loosely defined as either the absence of risk factors or factors that reduce the probability of a negative outcome (either through a direct pathway or via interaction with risk factors). Nevertheless, the focus of the extant literature on risk assessment has usually been on the identification of risk factors that are related to the risk of recidivism or future antisocial behavior. Much less attention has been devoted to the identification of protective factors that predict desistence from recidivism, in particular, the influence of individual and situational strengths on desistence (de Ruiter & Nicholls, 2011; Fougere & Daffern, 2011). As such, some scholars have argued that the exclusive focus of risk assessment measures on risk factors only may result in inaccurate assessment or judgments (Miller, 2006; Rogers, 2000). Importantly, there is recognition of the need to enhance the utility of violence risk assessment measures through identifying possible protective factors that may reduce the risk of recidivism (Farrington, 2007). The identification of protective factors will also help us better understand why some individuals desist from antisocial or offending behavior and can provide insights in terms of the development of prevention and intervention programs.

The extant literature on protective factors for antisocial and offending behaviors has been criticized on the lack of consistency regarding the definition of “protective factors.” Specifically, there is a lack of clarity as to whether the protective factors are conceptualized as “mirror images” of risk factors (i.e., the absence of the risk factors) or distinct entities (e.g., Borum, Bartel, & Forth, 2006; Luthar & McMahon, 1996). In addition, the inconsistencies surrounding the examination of the effects of protective factors, variable outcome measures, and failure to replicate results have undoubtedly contributed to the confusion in the interpretation of results across the field (Tolan, 2000). For example, protective factors are classified as either having a direct protective or buffering protective effect. The former relates to the protective factor being associated with a reduced probability of antisocial or offending behavior *regardless* of whether other risk factors are present. In contrast, buffering protective factors reduce the effects of the risk factor(s)—the key is the interaction effect between the *co-occurring* protective and risk factors.

Notwithstanding the confusion surrounding the conceptualization of protective factors and methodology used to examine the concept, there has been some progress in examining the protective factors for youth violence and delinquency in recent times. Preliminary results from a multi-site study suggests that factors relating to academic achievement, attachment to school, positive family management, as well as prosocial peer relationships may be important in reducing the onset of youth violence (Hall, Simon, Lee, & Mercy, 2012). Moreover, Lösel and Farrington (2012) described in their systematic review that a plethora of individual, family, school, peers, and neighborhood/community factors (e.g., above-average intelligence, low impulsivity/easy temperament, prosocial attitudes, close relationship to at least one parent, intensive parental supervision, sound academic achievement, nondeviant peers, living in a nondeprived and nonviolent neighborhood, etc.) were either direct or buffering protective factors for youth violence. Furthermore, Lösel and Farrington added that the probability of violence decreases as the number of protective factors increases, suggesting a dose–response relationship.

With regard to protective factors for sexual recidivism, there is a lack of research in this area. In Tharp et al.’s (2013) qualitative review of risk and protective factors for sexual aggression, they pointed out that potential protective factors for the onset of sexual violence might include having empathy for others, possessing good emotional health, being connected with others, academic achievement, as well as having parents who use reasoning to resolve conflict. Nevertheless, it is important to note that factors related to the onset of sexual aggression are not necessarily the same factors that are involved in the continued perpetuation of sexual offending behavior; hence, it is imperative to investigate those factors that are associated with the desistence of sexual recidivism in YSO.

### Measures for Assessing Protective Factors in Youth Offenders

Although there are many studies examining the utility of risk assessment measures for predicting recidivism, there are very few studies on the predictive utility of strengths or protective factors in youth offender risk assessment measures. Two youth offender risk assessment measures that include protective factors in addition to the risk factors for recidivism are the Structured Assessment of Violence Risk in Youth (SAVRY; Borum et al., 2006) and the Youth Level of Service/Case Management Inventory (YLS/CMI; Hoge & Andrews, 2011). With regard to the research on the utility of protective factors in these risk assessment measures, Rennie and Dolan (2010) found that the SAVRY protective factor total score was significantly predictive of desistence from general recidivism but not violent recidivism; in addition, *resilient personality traits* constituted the only significant individual protective factor.

In another study, Lodewijks, de Ruiter, and Doreleijers (2010) found that the SAVRY protective factors could buffer or mitigate the risk of violent recidivism. Moreover, these protective factors contributed to a significant incremental predictive validity for violent recidivism when added to a prediction model comprising of dynamic risk factors. Furthermore, in the medium to high-risk subgroups, the violent recidivism rate was significantly higher when protective factors were absent as compared with when protective factors were present. Results from Chu et al.’s (2014) large-scale study of youth offenders in a non-Western context revealed that several YLS/CMI strengths were univariately associated with desistence from general recidivism (*Family Circumstances/Parenting, Education/Employment*, and *Leisure/Recreation*). Furthermore, it was noted that these strengths did not necessarily reflect the absence of the risk factors, and that Education/Employment remained significant in a multivariate model for predicting general recidivism (inverse relationship) even after accounting for risk factors and other special considerations.

Pertaining to studies that have examined the relationship between protective factors and sexual recidivism, Schmidt, Campbell, and Houlding (2011) found that the SAVRY protective factor total score was not predictive of future sexual offenses in a sample of youth offenders, but the SAVRY protective factor total score was predictive of violent and nonviolent recidivism. Similarly, findings from Spice, Viljoen, Latzman, Scalora, and Ullman (2013) revealed that none of the SAVRY protective factors were significantly associated with desistence from sexual recidivism in a sample of YSO; *strong attachment bonds* was the only SAVRY protective factor that was inversely associated with nonsexual recidivism. These findings collectively suggest that there may be similar protective factors for nonsexual recidivism, but distinct protective factors for sexual recidivism among YSO.

Overall, there appears to be a paucity of measures that are designed specifically to assess protective factors within YSO. One measure that was designed for such a purpose was the *Protective Factors Scale* (Bremer, 1998), and it was designed to inform placement decisions for YSO; however, this measure has yet to be empirically validated. Another measure, the AIM2 has also been developed to holistically assess the strengths and concerns associated with youth who have sexually abused others; preliminary analyses have found the strengths scale to be associated with sexual recidivism (Griffin, Beech, Print, Bradshaw, & Quayle, 2008). More recently, two instruments have been developed to measure protective factors—the *Desistence for Adolescents Who Sexually Harm* (DASH-13), and the *Structured Assessment of Protective Factors for Violence Risk* (SAPROF). Worling (2013) has recently developed the DASH-13, which measures 13 (purported) protective factors that pertain to desistence of youth sexual offending. Similar to the Protective Factors Scale, no validation study has been published on the DASH-13 yet. The second instrument, the SAPROF, is a 17-item structured professional judgment measure that was initially developed to assess the protective factors associated with future risk of violence, but which may also be used to assess individuals who have sexually offended (de Vogel, de Ruiter, Bouman, & de Vries Robbé, 2012). It should be noted that the SAPROF was not specifically designed to measure protective factors in adolescents, but previous research has found the STATIC-99, a risk assessment instrument for adults, to possess adequate predictive validity among youth who have sexually offended (Viljoen, Mordell, & Beneteau, 2012). The SAPROF may be used in a similar manner to assess youth who have sexually offended, and the current article will thus seek to explore its use among the population.

### The Present Study

Considering that there is currently limited empirical knowledge pertaining to the protective factors for YSO, the present study sought to examine the utility of two measures of protective factors (i.e., the DASH-13 and the SAPROF) among YSO. In particular, the present study sought to examine whether the both these measures have utility in predicting the desistence of sexual and nonsexual recidivism in YSO. The following hypotheses were tested:

**Hypothesis 1:** The total score of the DASH-13 and SAPROF would be negatively correlated with the total score of a youth sexual offender risk assessment measure (i.e., the *Estimate of Risk of Adolescent Sexual Offense Recidivism* (ERASOR; Worling & Curwen, 2001).
**Hypothesis 2:** The total score of the DASH-13 and SAPROF would significantly predict sexual and nonsexual desistence from sexual recidivism in YSO.

## Method

### Source Sample

The current sample was derived from the same sample as that of Chu, Ng, Fong, and Teoh (2012). Pre-existing data on the ERASOR from that previous article was combined with current data on the DASH-13 and the SAPROF for the purposes of the present article. Therefore, the current sample consisted of 97 male youth (aged 12-18 years) who were referred to the Clinical and Forensic Psychology Branch (CFPB) of the Ministry of Social and Family Development (Singapore) between June 2003 and December 2007 for a psychological assessment of their risk of sexual recidivism and suitability for placement in a sexual offending treatment program. Their mean age at referral was 15.11 years (*SD* = 1.44).

Before referral to a psychologist, youth in the present study would have undergone an initial presentencing assessment by probation services for an index sexual offense or been found to have committed sexual offenses during their stay in a youth correctional institution. Specifically, more than half (58.8%, 57/97) of them were placed on community supervision, whereas the remaining 41.2% (40/97) were residing in a youth correctional institution at some point during their court orders. Of the total sample, three quarters (76.3%, 74/97) had committed molestation, 10.3% (10/97) rape, and 36.1% (35/97) other sexual offenses (e.g., nonconsensual fellatio, voyeurism, and indecent exposure).

### Procedure

The current study was retrospective in nature. Five psychologists from the Ministry conducted clinical file reviews, and completed the ERASOR, the DASH-13, and the SAPROF based on file information. Specifically, as mentioned, file review data for the ERASOR were derived from Chu et al. (2012), which were completed by three psychologists. In addition, file reviews for the DASH-13 and the SAPROF were conducted by two psychologists (G.Z. and Y.L.) for the current study. For the ERASOR, all raters were formally trained in the rating all three instruments, and possessed at least 1 year of using the ERASOR in assessment and research. For the SAPROF, only one rater (G.Z.) received training in the form of a half-day workshop conducted by the developers of the instrument; the other rater (Y.L.) did not receive any training but had discussions on coding with G.Z., and used the SAPROF manual and notes from the abovementioned training to assist with coding. For the DASH-13, both raters did not receive any formal training as there is as yet no formal training program for the use of the DASH-13. Prior to coding for the protective factors, both raters (G.Z. and Y.L.) agreed on the criteria for rating both the DASH-13 and the SAPROF. All raters were blind to the recidivism data of the youth sample. The clinical files contained (a) psychological reports prepared by psychologists at CFPB, (b) presentencing reports prepared by probation officers, (c) institution risk and criminogenic needs reports, (d) charge sheets, (e) statement of facts, and (f) school reports. Each file review yielded demographic and offense-related information, including personal, family, psychiatric, and criminal offending histories as well as current offending behaviors and risk management issues.

Pertaining to recidivistic outcomes, the current study examined both *sexual* (e.g., indecent exposure, molestation, peeping, rape, and sodomy) and *nonsexual recidivism* (i.e., any offenses that were not classified as sexual in nature), of both violent and nonviolent nature, that were committed by the youth during the follow-up. Recidivism was defined as the occurrence of an offense that resulted in a criminal charge, whereas desistence from the aforementioned recidivistic outcomes was operationalized as the absence of these outcomes. Recidivism data were coded by the second author (C.M.), who was blind to the DASH-13 and SAPROF ratings; the cut off for the recidivism follow-up was set as April 6, 2010.

### Ethical Approval

Approval for the current research study was obtained from the Ministry of Family and Social Development.

### Measures

#### DASH-13

The DASH-13 (Worling, 2013) is a structured checklist of possible factors that may contribute to desistence of adolescent sexual offending. Based largely on current understanding of protective factors of the onset (instead of continuance) of sexual violence, the DASH-13 is presently undergoing studies of its psychometric properties. Hence, its utility in enhancing the predictive validity of other established risk assessment measures has yet to be ascertained. The 13 protective factors fall under 2 domains—1 containing protective factors for pertaining specifically to desistence from sexual offending and the other consisting of factors relating to general functioning. The former (DASH-Sexual) consists of seven items: *prosocial sexual interests, prosocial sexual attitudes, prosocial sexual environment, awareness of the consequences of sexual reoffending, adequate environmental controls that match risk to reoffend sexually, hope for a healthy sexual future*, and *successful completion of sexual offense-specific treatment*. The latter (DASH-General) includes *compassion for others, positive problem-solving skills, positive affect-regulation skills, emotional intimacy with peers, close relationship with a prosocial supportive adult*, and *involvement in structured activity with prosocial peers*. The rating of each protective factor is done dichotomously (*Yes* or *No*), with an option to indicate whether there is not enough information to code an item. At the end of the assessment, the number of *Yes* responses can be totaled to give an overall score ranging from 0 to 13. The last item on successful treatment completion was automatically treated as an omitted item during analysis; in addition, the item relating to environmental controls was also removed during analysis as all youth in the sample were placed under a court order that would have placed risk-matched controls on the youth’s environment.

#### SAPROF

The SAPROF (de Vogel et al., 2012) constitutes a 17-item structured assessment guideline that is used to measure protective factors that may reduce the risk of violent or sexually deviant behavior. Similar to the DASH-13, validation of the SAPROF is ongoing, and its predictive validity with regard to promoting desistence especially with regard to violent and sexual offending has yet to be firmly established. Each item/factor is coded on a 3-point scale from 0 to 2, where “0” represents the absence of the protective factor, “1” indicates that the factor is present to some extent, and “2” signals a clear presence of the factor. A total protection score may then be returned from the sum of all item scores, but three sub-factors may also be derived from the SAPROF—(a) internal factors pertain to individual factors, (b) motivational factors that address the motivation to behave in a positive manner, and (c) external factors that refer to the positive environmental factors.

#### ERASOR

The ERASOR (Worling & Curwen, 2001) is an empirically guided, structured clinical judgment measure designed to assist clinicians in estimating the risk of sexual recidivism for youth (aged 12-18 years) who have presented with sexual offending behaviors. It comprises 25 items (16 dynamic and 9 static risk factors) that are grouped into five sections representing the 5 risk domains for sexual recidivism: (a) Sexual Interests, Attitudes, and Behaviors; (b) Historical Sexual Assaults; (c) Psychosocial Functioning; (d) Family/Environmental Functioning; and (e) Treatment. Items are coded as *Unknown*, *Not Present*, *Possibly/Partially Present*, or *Present*, where a score of “0” was assigned for items coded as *Unknown*, a score of “1” was assigned for items coded as *Not Present*, a score of “2” was assigned for items coded as *Possibly/Partially Present*, and a score of “3” was assigned for items coded as *Present*. No cutoff scores or formulas apply in determining the ERASOR risk level, instead evaluators make an overall clinical rating (i.e., structured professional rating/judgment) of *Low*, *Moderate*, or *High* risk. The current study examined domain and total scores that were derived from summing the scores for their respective items. However, items on the Treatment domain were excluded from all analyses because the youth in the sample had not received any treatment at the time of assessment. The ERASOR has been shown to have excellent reliability (e.g., Intraclass Correlation Coefficients [ICCs ]> .80 for total score and clinical judgment rating; Worling, Bookalam, & Litteljohn, 2012), and moderate predictive validity for predicting sexual recidivism (e.g., weighted Area Under Curve, AUC = .66 for both total score and clinical judgment rating; Viljoen et al., 2012). Importantly, it has been validated in the Singaporean context (Chu et al., 2012).

### Statistical Analyses

Descriptive statistics were first used to characterize the sample, with continuous data presented in terms of means and standard deviations. Pearson’s *r* correlations were conducted to examine the relationship between the total and domain scores of the DASH-13 and SAPROF and the total score of the ERASOR. Spearman’s *r* was also calculated to explore the relationship between the total and domain scores of the DASH-13 and SAPROF, and sexual and nonsexual recidivism.

In addition, Receiver Operating Characteristics (ROC) analyses were conducted to examine the predictive validity of the total and domain scores of the DASH-13 and the SAPROF. The ROC, which generates an AUC, is a commonly used method for examining the predictive validity of risk assessment measures, and it is less dependent on the base rates of reoffending than traditional measures of predictive accuracy (Douglas & Webster, 1999). Furthermore, Cox regressions were carried out to examine the incremental predictive validity of both the DASH-13 and the SAPROF for sexual and nonsexual recidivism over the ERASOR, while accounting for unequal time at risk. Specifically, the total score for the ERASOR was entered into Step 1, followed by either the DASH-13 or the SAPROF in Step 2. Analyses were conducted using SPSS Version 19.

## Results

### Inter-Rater Reliability

To examine the inter-rater reliability for the measures, the five raters separately coded a randomly selected sample of 16 (16.7%) files. The intra-class correlation coefficients for single rater (using absolute agreement definition; ICCs) were .49 (fair) for the ERASOR total score, .54 (fair) for the DASH-13 total score, and .65 (good) for the SAPROF total score (see Cicchetti, 1994 for a classification of ICCs). With regard to individual domains, ICCs were .64 (good) and .46 (fair) for the DASH-13 sexual and general protective factors, respectively, and .47 (fair), .71 (good), and .58 (fair) for the internal, motivational, and external domains of the SAPROF, respectively.

### Characteristics

The mean follow-up period was 1,637 days (*SD* = 491, range = 817-2,741), which was initiated from the date at which the youth were referred to CFPB. With regard to the recidivism rates, 7.2% (7/97) and 26.8% (26/97) of YSO reoffended sexually and nonsexually, respectively, during the follow-up period. The mean ERASOR score for the sample was 36.82 (*SD* = 6.17, range = 24-52), whereas the mean DASH-13 total score was 5.12 (*SD* = 2.29, range = 0-10) and the mean SAPROF total score was 12.49 (*SD* = 3.89, range = 0-23).

### Relationship Between Protective and Risk Factors and Recidivism

To examine the relationship between protective and risk factors, Pearson’s *r* was conducted between scores on the DASH-13 and SAPROF and the ERASOR. From Table 1, as hypothesized, results indicated that both the total scores for the DASH-13 and SAPROF, as well as scores for their domains (DASH-Sexual, DASH-General, SAPROF-Motivational, SAPROF-External) were inversely related to the ERASOR total score, indicating that a higher presence of protective factors was associated with lower levels of sexual reoffending risk.

**Table 1.** Correlation of DASH-13, SAPROF, and ERASOR Scores With Sexual and Nonsexual Reoffending.

	Sexual recidivism	Nonsexual recidivism	DASH-Sexual	DASH-General	DASH-Total	SAPROF-Internal	SAPROF-Motivational	SAPROF-External	SAPROF-Total	ERASOR–Sexual interests	ERASOR-Historical	ERASOR-Psychological	ERASOR-Family	ERASOR-Total
DASH-Sexual	−.02	−.21*	—											
DASH-General	−.13	−.18	.44**	—										
DASH-Total	−.09	−.20*	.86**	.83**	—									
SAPROF-Internal	.05	−.13	.45**	.70**	.67**	—								
SAPROF-Motivational	.00	−.19	.50**	.66**	.68**	.55**	—							
SAPROF-External	−.02	−.03	.28**	.32**	.35**	.36**	.32**	—						
SAPROF-Total	.02	−.18	.55**	.74**	.75**	.77**	.91**	.59**	—					
ERASOR–Sexual interests	.09	.26**	−.54**	−.18	−.44**	−.15	−.14	−.04	−.15	—				
ERASOR-Historical	.26**	.10	−.38**	−.15	−.31**	−.01	−.10	−.05	−.07	.56**	—			
ERASOR-Psychological	.02	.09	−.03	−.22*	−.14	−.18	−.15	−.01	−.15	.25*	−.07	—		
ERASOR-Family	−.08	.23*	−.03	−.22*	−.15	−.23*	−.22*	−.20	−.27**	.05	.09	.19	—	
ERASOR-Total	.18	.24*	−.40**	−.32**	−.43**	−.20	−.28*	−.10*	−.25**	.73**	.68**	.57**	.34**	—

*Note.* DASH = Desistence for Adolescents Who Sexually Harm; SAPROF = Structured Assessment of Protective Factors for Violence Risk; ERASOR = Estimate of Risk of Adolescent Sexual Offense Recidivism.

* *p* < .05. **p < .01.

Next, the relationship between protective factors and recidivism was examined by correlating scores on the DASH-13 and SAPROF with both sexual and nonsexual recidivism (dichotomous variables). As presented in Table 1, Spearman’s *r* indicated that the DASH-Total, DASH-Sexual, and DASH-General were not significantly correlated to sexual recidivism. However, the DASH-Total was inversely related to nonsexual recidivism, whereas surprisingly, the DASH-Sexual was inversely related to nonsexual recidivism. Neither the SAPROF-Total nor any of its domains were related to sexual or nonsexual recidivism.

### Predictive Validity for Desistence and Recidivism

AUCs were calculated to first examine the predictive validity of the DASH-13 and the SAPROF for sexual and nonsexual desistence, and for the ERASOR for sexual and nonsexual recidivism. In general, AUCs of .56, .64, and .71 may be considered to be of small, medium, and large effect sizes, respectively (Rice & Harris, 2005). As presented in Table 2, using the full follow-up period, AUCs for both the DASH and the SAPROF were nonsignificant for desistence from sexual recidivism. With regard to nonsexual recidivism, AUCs for the DASH-sexual and DASH-total scores were equivalent to small effect sizes. In addition, the ERASOR-Historical domain had an AUC equivalent to a large effect size with regard to sexual recidivism; the ERASOR–Sexual interests, ERASOR-Family, and ERASOR total scores had AUCs equivalent to medium effect sizes with regard to nonsexual recidivism.

**Table 2.** The Predictive Validity of the DASH, SAPROF, and ERASOR for Desistence From Sexual and Nonsexual Recidivism.

Type	Domain	AUC	Effect size	95% CI	*p*
Follow-up (up to 2,741 days)					
Sexual desistence	DASH-Sexual	.52	Small	[.35, .70]	*ns*
	DASH-General	.64	Medium	[.44, .84]	*ns*
	DASH-Total	.60	Small	[.45, .75]	*ns*
	SAPROF-Internal	.45	Small	[.19, .70]	*ns*
	SAPROF-Motivational	.51	Small	[.30, .71]	*ns*
	SAPROF-External	.52	Small	[.34, .70]	*ns*
	SAPROF-Total	.48	Small	[.28, .69]	*ns*
Nonsexual desistence	DASH-Sexual	.63	Small	[.50, .76]	.049
	DASH-General	.62	Small	[.49, .75]	*ns*
	DASH-Total	.63	Small	[.50, .76]	.048
	SAPROF-Internal	.58	Small	[.45, .71]	*ns*
	SAPROF-Motivational	.63	Small	[.50, .76]	*ns*
	SAPROF-External	.52	Small	[.39, .65]	*ns*
	SAPROF-Total	.62	Small	[.49, .75]	*ns*
Sexual recidivism	ERASOR–Sexual interests	.60	Small	[.40, .81]	*ns*
	ERASOR-Historical	.79	Large	[.67, .91]	.011
	ERASOR-Psychological	.52	Small	[.35, .69]	*ns*
	ERASOR-Family	.42	Small	[.23, .62]	*ns*
	ERASOR-Total	.70	Large	[.53, .87]	*ns*
Nonsexual recidivism	ERASOR–Sexual interests	.67	Medium	[.54, .80]	.011
	ERASOR-Historical	.56	Small	[.44, .69]	*ns*
	ERASOR-Psychological	.56	Small	[.42, .69]	*ns*
	ERASOR-Family	.65	Medium	[.53, .76]	.029
	ERASOR-Total	.66	Medium	[.53, .78]	.019

*Note.* DASH = Desistence for Adolescents Who Sexually Harm; SAPROF = Structured Assessment of Protective Factors for Violence Risk; ERASOR = Estimate of Risk of Adolescent Sexual Offense Recidivism; AUC = Area Under Curve; *ns* = nonsignificant.

To account for unequal follow-up periods, ROC analyses were also performed using a fixed follow-up period of 2 years (730 days). Using this follow-up period, none of the AUCs for the SAPROF and DASH-13 were significantly predictive of desistence, nor were the AUCs for the ERASOR significantly predictive of the recidivism outcomes (these results are available on request from the first author). However, it should be noted that only 0.01% (1/97) and 3.09% (3/97) of YSO reoffended sexually and nonsexually, respectively, during the follow-up period, and these reduced base rates may account for these results.

Cox regressions were then conducted to examine the incremental predictive validity of the DASH-13 and SAPROF over the ERASOR for sexual and nonsexual recidivism, while accounting for unequal time at risk (see Tables 3 and 4). Results indicated that the ERASOR did not significantly predict sexual or nonsexual reoffending, and neither the DASH-13 nor the SAPROF were significant predictors in these models either.

**Table 3.** Incremental Validity of the DASH-13 and the SAPROF Over the ERASOR for Sexual Recidivism.

	*B*	*SE*	Wald	*df*	*p*	Exp(*B*)	95% CI	−2 log likelihood
Step 1								638.141
ERASOR total score	−.022	.016	2.004	1	.154	0.978	[0.949, 1.009]	
Step 2								637.914
ERASOR total score	−.026	.018	2.129	1	.145	0.974	[0.940, 1.009]	
DASH-13 total score	−.025	.053	0.229	1	.633	0.975	[0.879, 1.081]	
Step 1								638.141
ERASOR total score	−.022	.016	2.004	1	.154	0.978	[0.949, 1.009]	
Step 2								637.830
ERASOR total score	−.025	.017	2.273	1	.132	0.975	[0.944, 1.008]	
SAPROF total score	−.018	.033	0.307	1	.580	0.982	[0.921, 1.047]	

*Note.* DASH = Desistence for Adolescents Who Sexually Harm; SAPROF = Structured Assessment of Protective Factors for Violence Risk; ERASOR = Estimate of Risk of Adolescent Sexual Offense Recidivism.

* *p* < .05.

**Table 4.** Incremental Validity of the DASH-13 and the SAPROF Over the ERASOR for Nonsexual Recidivism.

	*B*	*SE*	Wald	*df*	*p*	Exp(*B*)	95% CI	−2 log likelihood
Step 1								484.981
ERASOR total score	−.016	.018	0.705	1	.401	0.985	[0.950, 1.021]	
Step 2								484.433
ERASOR total score	−.022	.020	1.137	1	.286	0.979	[0.940, 1.018]	
DASH-13 total score	−.046	.062	0.558	1	.455	0.955	[0.845, 1.078]	
Step 1								484.981
ERASOR total score	−.016	.018	0.705	1	.401	0.985	[0.950, 1.021]	
Step 2								483.648
ERASOR total score	−.019	.019	1.034	1	.309	0.981	[0.945, 1.018]	
SAPROF total score	−.044	.039	1.305	1	.253	0.957	[0.887, 1.032]	

*Note.* DASH = Desistence for Adolescents Who Sexually Harm; SAPROF = Structured Assessment of Protective Factors for Violence Risk; ERASOR = Estimate of Risk of Adolescent Sexual Offense Recidivism.

* *p* < .05.

## Discussion

The current article constitutes an examination into the protective factors of YSO in a non-Western (Singaporean) context, as well as an investigation of the utility of a specific desistence measure for YSO. As such, it seeks to contribute to the limited available research by investigating the associations and predictive validity between the protective factors listed in the DASH-13 and SAPROF and desistence from recidivism.

With regard to recidivism, the present study found that higher numbers of protective factors in the sexual domain of the DASH-13 was related to better desistence from nonsexual reoffending, suggesting that purported factors that pertain specifically to sexual reoffending may also protect YSO from further committing nonsexual offenses. This is important because it has been shown that the majority of YSO that recidivate go on to commit nonsexual instead of sexual offenses (Caldwell, 2007, 2010; Chu & Thomas, 2010). However, an increase in the number of protective factors present was not related to lower incidences of sexual recidivism among YSO. Previous studies with similar base rates have also not found such an association between protective factors and sexual recidivism (Spice et al., 2013). In addition, neither the total nor the domains scores of the SAPROF were significantly associated with both sexual and nonsexual recidivism. This is in contrast to studies which have found the SAPROF to correlate with sexual and nonsexual recidivism (de Vries Robbé, de Vogel, & de Spa, 2011; de Vries Robbé, de Vogel, & Stam, 2012; de Vries Robbé, de Vogel, & Douglas, 2013).

The findings with regard to predictive validity did not provide support for the second hypothesis. In contrast with previous findings (de Vries Robbé et al., 2011; de Vries Robbé et al., 2013), neither the total nor domain scores of the DASH-13 and the SAPROF produced acceptable levels of predictive validity with regard to sexual and nonsexual desistence. In fact, on the basis of current findings, caution should be exercised when using these instruments to predict desistence from sexual or nonsexual recidivism. For the ERASOR, current results indicate that the instrument is predictive of recidivism, adding to the findings of previous studies that have consistently demonstrated acceptable predictive validity for sexual recidivism (Chu et al., 2012; Worling et al., 2012). In addition, the investigation of incremental predictive validity suggests that neither the DASH-13 nor the SAPROF added predictive utility to the ERASOR. These findings contrast with those of others where incremental predictive validity has been reported for the SAPROF over measures of violence risk assessment (e.g., Historical, Clinical, Risk Management-20 [HCR-20]: de Vries Robbé et al., 2011; de Vries Robbé et al., 2013).

One possible partial explanation for the current results may be the low base rate of sexual reoffending in the current sample of YSO. Using the full follow-up period, only seven youth sexually reoffended; this decreases to just one youth who sexually reoffended when using a fixed follow-up period (730 days) that represented an equal time at risk of the entire sample. In part, such a low base rate is linked to the low sample size for the current study, which would have affected the power of some analyses. Although it is encouraging that so few youth have been referred for assessment of risk of sexual recidivism in the 5 years from 2003 to 2007, and that even fewer have sexually reoffended, such low numbers may have contributed to difficulty in detecting any significant association between the current measures and sexual recidivism, and also affect the accuracy with which desistence or recidivism may be predicted (Caldwell, 2010).

The intra-class coefficients for all three measures used here were also relatively low, as compared with those reported by the developers of these measures. This may imply that these instruments may not have been scored or rated consistently, at least for the current study. Such inconsistency may have in turn affected the validity of the results. However, it must be noted that this was a retrospective study that relied on archival data collected for the purpose of assessment and intervention, instead of that tailored for the specific aims of this study. Future studies could thus examine protective factors in a prospective context (i.e., through face-to-face interviewing), whereby the specific required information with regard to such factors may be derived not only to a greater extent, but with greater reliability.

Another possible reason could relate to the nature (and not number) of protective factors that serve to protect YSO against sexual recidivism. For example, fearing the loss of one’s social integrity and feeling shame has been found to be a protective factor against sexual aggression, specifically among Asian American men as compared with European American men (Nagayama Hall, Teten, DeGarmo, Sue, & Stephens, 2005). Given that the current sample was made up of male youth from Singapore, there may be other unexplored but culturally specific protective factors that are related to sexual recidivism.

The characteristics of the current instruments may have also contributed to current results. Both the DASH-13 and the SAPROF were not designed to assess protective factors with regard to general reoffending outcomes—the DASH-13 and SAPROF were designed to assess protective factors associated with desistence from sexual or violent reoffending. Both instruments may therefore not be sensitive to factors for desistence from nonsexual reoffending. In particular, the SAPROF has been designed for, and only been validated with adults. Certain items within the SAPROF may be more relevant for adults than youth (e.g., Item 8: Financial Management; Item 11: Positive Life Goals; Item 14: Intimate Relationship). This may affect the ability of the SAPROF in providing an overall picture of protective factors for youth. However, a youth version of the SAPROF is under development, and could be further validated to investigate the protective factors of YSO.

The use of retrospective file information at the time of referral also meant that the results of any targeted intervention and treatment that was subsequently offered to the youth in the study could not be taken into account in terms of both sexual and nonsexual recidivism, potentially also affecting the accuracy of the predictive validity for each measure. In addition, given the retrospective nature of the study, we were not able to determine the time (and so deduct it from the follow-up period) for which almost half of the sample (41.2%) who resided within a youth correctional institution during their court orders were at risk. Although the full sample was at risk to reoffend during the follow-up period, those YSOs subject to types of custodial placements may have been at risk of reoffending for shorter periods.

Finally, reliance on official records of recidivism would most likely have resulted in under-reporting of sexually abusive behavior or other recidivism that may not have been detected. Certainly, further research is required to gather additional empirical evidence on the validity of both the DASH-13 and SAPROF. Although current results do not support the predictive validity of either instrument, there may still be some utility in using the DASH-13 (and a youth-adapted version of the SAPROF) for individual case management and intervention, where building on protective factors may assist rehabilitation through the mitigation of criminogenic risk factors and needs (Lodewijks et al., 2010). In fact, the SAPROF was designed to be used to assess adults in conjunction with a structured professional judgment tool such as the Historical Clinical Risk management: Version 3 to produce a final risk judgment which includes an assessment of both protective and risk factors (de Vogel et al., 2012; de Vries Robbé et al., 2012). Final risk judgments could not be produced in the current study, as the ERASOR was rated by a different set of raters (from Chu et al., 2012) as the DASH-13 and SAPROF, but these should be investigated in future studies.

Similarly for case management, deploying measures of protective factors alongside measures of risk assessment among youth who have sexually offended may provide a more balanced overview of not only the factors which may contribute to recidivism, but also those which may encourage desistence (de Vogel et al., 2012; Miller, 2006).

Chu et al. (2014) identified three domains (family circumstances, education/employment, and leisure/recreation) of the YLS/CMI that were associated with general recidivism (which includes sexual recidivism), and have suggested that protective and risk factors may be involved in more complex and indirect relationships that predict recidivism (Chu et al., 2014). For instance, the effect of risk factors on recidivism may be buffered (moderated) in the presence of protective factors, and protective factors could encourage desistence among the YSO through the lowering of the risk of recidivism. Such relationships should be examined in future studies.

Indeed, prospective longitudinal studies may also be able to provide information on the patterns of change in desistence in relation to protective factors across time (Lösel & Farrington, 2012). In fact, the causal mechanisms through which protective factors are able to influence desistence among YSO may also be determined to a greater extent within a longitudinal design (Lösel & Farrington, 2012); such research would contribute to the way in which protective factors may be used to increase the chances of desistence from recidivism in youth who have sexually offended. Intervention may then seek to target the risks identified above, while also focusing on building stronger and closer relationship with protective factors that could play a part in promoting desistence. Assessments that are able to identify both risk and protective factors may thus prove to be beneficial in the delivering of service and intervention to youth who have offended.

The current study represents only a preliminary examination of the protective factors among the YSO in Singapore. Although results here suggest that caution should be exercised while using both the DASH-13 and the SAPROF among YSO, follow-up studies that address the limitations and considerations raised here are warranted.
